# Optimal or not; depends on the task

**DOI:** 10.3758/s13423-018-1536-4

**Published:** 2018-11-08

**Authors:** Nathan J. Evans, Aimée J. Bennett, Scott D. Brown

**Affiliations:** 1 Department of Psychology, University of Amsterdam, Amsterdam, The Netherlands; 2 School of Psychology, University of Newcastle, Callaghan, Australia

**Keywords:** Decision-making, Reward rate, Optimality, Task demands, Bayes factors

## Abstract

Decision-making involves a tradeoff between pressures for caution and urgency. Previous research has investigated how well humans optimize this tradeoff, and mostly concluded that people adopt a sub-optimal strategy that over-emphasizes caution. This emphasis reduces how many decisions can be made in a fixed time, which reduces the “reward rate”. However, the strategy that is optimal depends critically on the timing properties of the experiment design: the slower the rate of decision opportunities, the more cautious the optimal strategy. Previous studies have almost uniformly adopted very fast designs, which favor very urgent decision-making. This raises the possibility that previous findings—that humans adopt strategies that are too cautious—could either be ascribed to human caution, or to the experiments’ design. To test this, we used a slowed-down decision-making task in which the optimal strategy was quite cautious. With this task, and in contrast to previous findings, the average strategy adopted across participants was very close to optimal, with about equally many participants adopting too-cautious as too-urgent strategies. Our findings suggest that task design can play a role in inferences about optimality, and that previous conclusions regarding human sub-optimality are conditional on the task settings. This limits claims about human optimality that can be supported by the available evidence.

## Introduction

Substantial effort has been invested in understanding the optimality of perceptual decision-making (see Rahnev and Denison, 2018 for a review). These investigations have examined decisions about consumer choice (Tversky, 1972), the reported confidence for decisions (Baranski & Petrusic, 1994), the sequence of presented stimuli (Cheadle et al., 2014), and even life-or-death situations (Tversky & Kahneman, 1981). One kind of optimality that is relevant for speeded decision-making focuses on “reward rate”, which is the number of correct decisions that are made per unit of time (Drugowitsch et al., 2012; Bogacz et al., 2006; Evans & Brown, 2017; Starns & Ratcliff, 2012; Simen et al., 2009; Balci et al., 2011; Starns & Ratcliff, 2010). Optimizing reward rate implies making the greatest number of correct choices in the available time, and this requires a precise balance between caution and urgency. Making decisions too cautiously will result in a sub-optimal reward rate, as the rewards will take too long to obtain. Making decisions too urgently will also result in suboptimal reward rate, as the increased errors associated with urgency mean that fewer rewards can be obtained.

Previous work has shown that animals are sometimes able to identify the optimal policy to maximize reward rate (Chittka et al., 2003; Uchida & Mainen, 2003; Hawkins et al., 2015). In contrast, humans mostly appear not to maximize reward rate, instead adopting too-cautious strategies (Starns & Ratcliff, 2012; Evans & Brown, 2017; Bogacz et al., 2010; Starns & Ratcliff, 2010). Some studies have found humans to adopt strategies that are only slightly sub-optimal – but still too cautious – usually after training or other assistance (Simen et al., 2009; Balci et al., 2011; Evans & Brown, 2017; Starns & Ratcliff, 2010). The conclusion that participants, on average, prefer an overly cautious approach has spurred the development of theories to account for this finding. For example, Bogacz et al., (2006) investigated the asymmetry of rewards, and found that too-cautious responding usually presented a lower risk than too-urgent responding. Maddox and Bohil (1998) investigated whether participants might focus on a mixture of error minimization and reward rate maximization, rather than just the latter. Evans and Brown (2017) tested the theory that participants were simply unaware of the improvements in reward rate that could come from more urgent strategies.

We investigate a different type of explanation for the common finding of humans being sub-optimally cautious: that previous findings may have been biased by their task design. Most previous studies have shared a common methodological element: short delays from one decision to the next (see Table 1). Although this may seem like a minor methodological detail, the between-trial timings have a substantial impact on which strategy is optimal (Simen et al., 2009), and the behavior of humans (Jentzsch & Dudschig, 2009; Krueger et al., 2017). At the extreme limit, as the time between decisions tends towards zero, more and more urgent decisions with less and less caution will lead to more and more rewards per unit time, even if the decisions were no more accurate than chance. On the other hand, if the time between decisions is very long, the extra decision time added by increased caution represents a smaller relative cost (see also Eq. 1). We hypothesize that previous conclusions of humans being sub-optimally cautious may have been the result of the short delays requiring a strategy more urgent than participants could, or were willing to, implement.

**Table 1:** Properties and findings of key studies of reward rate optimality

Study	RSI_*c*_ (sec)	RSI_*e*_ (sec)	Difficulties	Relevant findings
Simen et al., (2009)	0.5/1/2^*a*^	0.5/1/2^*a*^	Single	Ps were approximately optimal
				for the longest RSI, and too
				cautious for shorter RSIs.
Bogacz et al., (2010)	0.5/1/2/0.5^*b*^	0.5/1/2/2^*b*^	Both^*c*^	$\sim $30% of Ps were close to
				optimality, though others were
				too cautious. Ps were closer
				to optimality with larger RSI.
Starns and Ratcliff (2010)	0.25-0.75^*d*^	0.55-1.05^*d*^	Multiple	Young Ps were closer to optimal
				than older Ps. Young Ps were
				close to optimal, though overall,
				both groups were too cautious.
Balci et al., (2011)	1^*e*^	1^*e*^	Multiple	Ps were approximately optimal
				in easier conditions, though too
				cautious in harder conditions.
Starns and Ratcliff (2012)	0.4	0.4	Multiple	Young Ps were too cautious in
				most conditions, old Ps were
				always too cautious.
Evans and Brown (2017)	0.4	0.9	Single	Ps were close to optimal with
				guidance, though were still too
				cautious, and this over-caution
				was greater without guidance.
Current Study	0.8	2.6	Single	Ps were optimal on average,
				with approximately equal
				numbers of Ps being too
				cautious and too urgent.

^a^ RSI differed between blocks, and were random between trials according to a normal distribution with a standard deviation of 0.1 s;

^b^ These experiments used an error timeout in the final condition;

^c^ Exp1 used a single difficulty, whereas Exp2 used multiple difficulties;

^d^ Timings were variables between different experiments;

^e^ RSI differed between blocks, and were random between trials according to an exponential distribution

All of these have, at least to some extent, concluded that participants were more cautious than optimal on average. We include our study in the final row as a comparison point, which found participants to be optimal on average. For each study, the columns show: the response-to-stimulus interval (RSI) following correct decisions (*RSI*_*c*_), the RSI following incorrect decisions (*RSI*_*e*_), whether the study used a single level of decision difficulty or mixed multiple levels of difficulty, and lastly, the relevant findings to our arguments

Our hypothesis is consistent with some of the findings of two previous reward rate studies: Simen et al., (2009), who directly investigated the effects of the experiment’s timing properties on reward rate optimality, and Bogacz et al., (2010), who manipulated the experiment’s timing properties, but mostly made general inferences about whether or not participants performed the task in an overall optimal manner. One of the experiments reported by Simen et al., (2009) manipulated the response to stimulus interval (RSI), which is the time delay between a participant making a response for one decision and the onset of the next trial. Using group-averaged data, Simen et al. found that with long RSIs decision-making was close to optimal; but still, on average, more cautious, and not more urgent. We aim to take this result further, and identify whether human decision-making can be optimal on average, or even overly urgent, if the task was set up appropriately. We design our task to push the optimal strategy towards caution even more than that of Simen et al., by differentially adding delays to the RSI following correct vs. incorrect decisions; a manipulation that was also used by Bogacz et al., (2010). Importantly, extra delays following incorrect decisions reward cautious decision-making Bogacz et al., and unlike standard RSI manipulations, do not force participants to wait after every trial. This allowed us to extend the incorrect decision RSI 30% longer than the longest used in the previous optimality studies (Table 1), without greatly reducing the number of trials that could be completed in a short experimental session, or risking participant disengagement from consistently long RSIs. If the conclusions of previous work are correct, and humans prefer too-cautious strategies, then we would expect to once again observe too-cautious decision-making in our experiment. On the other hand, if our hypothesis is correct and the design of previous tasks created an optimal strategy that was too urgent for humans to achieve, then it is possible that decision-making in our task with additional delays for error responses could be optimal, or even too urgent. We address the statistical inference side of this question using state-of-the-art methods, using Bayesian hierarchical methods (as with Evans and Brown 2017), and a novel comparison between optimal and non-optimal models via Bayes factors. These inferences have been made possible by recent advances in the estimation of marginal likelihoods for cognitive models (Evans and Annis, 2018).

## Method

### Participants

Eighty psychology students from the University of Newcastle completed the experiment online, which is a larger sample (per condition) than most studies in the literature have used (e.g., Bogacz et al., 2006; Simen et al., 2009; Starns and Ratcliff, 2012; Evans and Brown, 2017). Following Evans and Brown (2017), we removed data from one participant who failed to comply with the task instructions: that participant answered fewer than 70% of decisions correctly.

### Task and procedure

Participants made perceptual decisions about the motion direction of a random dot kinematogram, implemented with the “white noise” algorithm (Pilly & Seitz, 2009). Participants were required to decide whether a cloud of 40 white dots on a black background was moving towards the top-left (‘z’ key) or top-right (‘/’ key) of the screen, with the actually direction being randomly presented on each trial. The dots remained within a circular area, 150 pixels in diameter, in the center of the screen. Any dot leaving this area was randomly replaced within it. On each frame (66.7 ms), eight dots (20%) were randomly chosen to move $\sqrt []{18}$ pixels in the coherent direction for the current trial, and all other dots were randomly replaced within the circular area. After each correct decision, participants were shown the word “CORRECT” for 400 ms. After each incorrect decision, they were shown the word “INCORRECT” for 2200 ms. Any responses that were too fast to have been from actual decision-making processes (< 100 ms) resulted in a 2500-ms timeout, with “TOO FAST” appearing on the screen. After the feedback display, a blank screen was shown for 400 ms, after which the next trial commenced.

Participants completed 20 blocks of 60 trials each. Although previous research has commonly focused on “fixed-time” paradigms, where each block runs for a fixed amount of time and participants attempt to maximize their correct responses, Evans and Brown (2017) found that participants came closer to reward rate optimality in “fixed-trial” conditions, when given instructions that made the explicit goal to maximize reward rate. Following this, we instructed participants that each correct response that they made was worth one point, and that their goal in the experiment was to obtain as many points in each 1-min period of time (i.e., maximizing their reward rate).

### Design and data analysis

We excluded trials with responses that were considered to be too fast to have come from decision-making processes (< 100 ms), or so slow that people likely had lost attention during the trial (> 5000 ms). These exclusions eliminated approximately 0.3% of the data. Reward rate is the expected number of correct decisions per unit time, which can be calculated by Eq. 1:

1 $$ \frac{PC}{MRT + ITI + FDT + (1-PC)*ET} $$

Here *PC* is average accuracy, *MRT* is mean response time, *ITI* is the blank screen time after feedback (in our experiment, 400 ms), *FDT* is the feedback display time common to all trials (i.e., 400 ms), and *ET* is the error timeout added to *FDT* following incorrect trials (i.e., 1800 ms).

We used the diffusion model (Ratcliff, 1978) to estimate changes in reward rate for different potential speed–accuracy tradeoff strategies. The diffusion model is part of a general class of models known as evidence accumulation models (EAM; see Ratcliff, Smith, Brown, and McKoon, 2016, for a review, and van Ravenzwaaij, Dutilh, and Wagenmakers, 2012; Brown, Marley, Donkin, and Heathcote, 2008; Evans, Rae, Bushmakin, Rubin, and Brown, 2017; Evans, Hawkins, Boehm, Wagenmakers, and Brown, 2017 for applications), which propose that people make decisions by accumulating evidence from the environment for the different alternatives at some rate (known as the “drift rate”), until this evidence reaches a threshold amount of evidence (known as the “decision threshold”), and a response is triggered. We used the “simple” diffusion model^1^, which includes two additional parameters: the starting amount of evidence prior to accumulation (known as the “starting point”), which reflects a priori response biases, and the amount of time required by other processes involved in responding (known as “non-decision time”), such as perceptual and motor processing. The model operationalizes speed–accuracy tradeoff via the threshold parameter. Higher thresholds indicate more cautious responding, as more evidence is accumulated before a response is triggered, whereas lower thresholds indicate more urgent responding, as little evidence is required to trigger a response. The optimal strategy was defined as the threshold setting that lead to the highest reward rate (conditional on all other parameters being fixed at their estimated values). We identified the optimal threshold through a grid search of all possible threshold values between 0.01 and 4 in increments of 0.01, and using the closed form solutions of Bogacz et al., (2006) to calculate the expected error rate and mean response time for each possible threshold setting.

We performed two key analyses to assess whether, and if so how, participants’ threshold settings differed from optimality. Both analyses involved fitting the diffusion model to the empirical data, which we did through Bayesian hierarchical methods (Shiffrin, Lee, Kim, and Wagenmakers, 2008; see Evans and Brown 2018 for a discussion), and likelihood functions extracted from the *fastdm* package (Voss and Voss, 2007). Bayesian methods of estimation involve estimating a distribution of possible values for the parameters (known as the “posterior distribution”), which captures the uncertainty in the value of the parameter, as opposed to other methods that only obtain the most likely value of the parameter (e.g., maximum likelihood). Hierarchical models involve estimating the parameters of each individual participant while also constraining their parameter values to follow some group-level distribution, providing mutual constraint between the parameter estimation of different individuals.

Our first analysis follows Evans and Brown (2017), where we estimated the diffusion model with a different threshold parameter for each block. This additional freedom in the model allowed for the possibility that participants adjusted their threshold over blocks in response to their performance and feedback. Our key interest was in the estimated group-level distribution of threshold for each block, which we compared to an “optimal distribution” for each block, computed using the joint group-level posterior of the other three parameters of the model. We also performed these same analyses separately for each individual, to see whether these patterns appeared to be consistent across our entire sample.

Our second analysis used a novel model-based contrast to test whether participants were using the reward rate optimal strategy, and, if not, how they differed from optimality. We estimated four versions of the diffusion model, beginning with the regular diffusion model described above. The second version was the same, but had the threshold parameter constrained to the reward rate optimal value. The third model version constrained the threshold parameter to be greater than the optimal setting (i.e., a “too-cautious” model), and the fourth version constrained the threshold to be lower than the optimal setting (i.e., a “too-urgent” model). We compared these four models using their marginal likelihoods (i.e., Bayes factors), which were calculated with the TIDEz algorithm (Evans & Annis, 2018). This algorithm uses the Bayes factor calculations of thermodynamic integration (Annis et al., 2018; Friel & Pettitt, 2008) integrated with DE-MCMC (Turner et al., 2013) under the logic of thermodynamic integration through population MCMC (Calderhead & Girolami, 2009).

## Results

### Group-level analyses

**Fig. 1:**
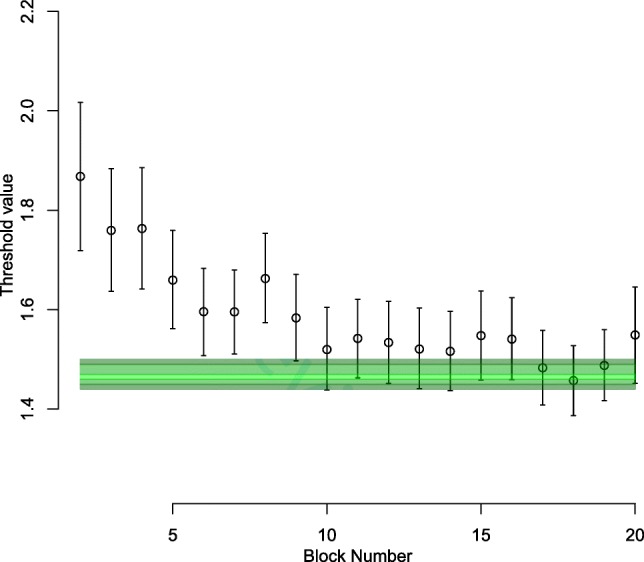
Posterior distribution over the population-level threshold parameter, compared with its optimal setting, over different blocks of trials. *Black circles* indicate the median of the threshold *μ* posterior, and *error bars* give the 2.5% and 97.5% quantiles. The *green region* is the distribution over the optimal threshold, calculated from the joint posterior of the values of the other parameters. The *brightest regions of green* show the center of the distribution (40% to 60% quantiles), and the more *transparent regions* being the tails (10 to 20% and 80 to 90% quantiles). Thresholds move towards the optimal region over blocks, eventually ending up within the optimal region

Figure 1 illustrates the results of our first analysis, focusing on the group-level estimates of the threshold parameter for each block of the experiment. The dots display the posterior median of the group-level threshold parameter, with error bars. The green region shows the posterior distribution for the optimal setting of the threshold parameter. These results are in agreement with those of Evans and Brown (2017), although there is now greater precision due to the increased sample size in the current experiment. In the first blocks of the experiment (i.e., blocks 2-5), participants were more cautious than optimal for maximizing reward rate. Over time, their threshold settings move closer to the optimal region (in line with the findings of Starns and Ratcliff, 2010; Balci et al., 2011; Evans and Brown, 2017). After block 10, the thresholds appear to be fairly consistent until the end of the experiment, and are close to the optimal region. Interestingly, Evans and Brown (2017) found the group with the level of feedback used in our experiment (labeled “Info” in their study) to begin to approach optimality, there was still some distance between their adopted threshold and the optimal threshold. The participants in our experiment, with slower trial-to-trial timing, appear to have achieved optimality better than those studied by Evans and Brown (2017).

Our second analysis compared the four models using Bayes factors. For this, the models were estimated using data from blocks 11–20, during which behavior was approximately stationary. When data from all participants were treated together, hierarchically, the log-marginal likelihood was highest for the non-optimal, freely estimated threshold model (10,594), followed by the too-cautious model (10,222), then the too-urgent model (10,054), and lastly the optimal model (9965). These findings indicate that (1) despite participants approaching optimality and occasionally being within the optimal region, there is decisive evidence in favor of participants being suboptimal, based on the non-optimal models all beating the optimal model, (2) participants seem to be more on the too-cautious side than the too-urgent side, as the too-cautious model was preferred over the too-urgent model, and (3) the preference for too-cautious responding may have been inconsistent over participants, as the completely free threshold was preferred over both the too-cautious and too-urgent models. This last conclusion is important, as it suggests that people were not, on average, too cautious.

### Individual-level analyses

**Fig. 2:**
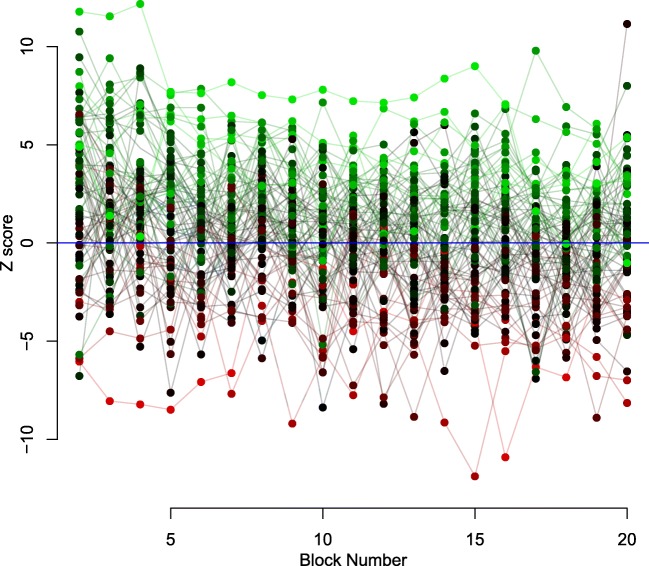
Estimated thresholds compared to optimal settings, for each individual participant. Dots show Z scores calculated for each block, and for each participant on the difference between the estimated threshold posterior and the optimal threshold distribution, with the optimal threshold distribution calculated based on the joint posterior of the other parameters for that individual participant. The *lines* connect the subjects across blocks. The color of the dots and lines differs between subjects, and reflects each subject’s average difference from optimality across all blocks. *Green dots*/*lines* show participants who were on average extremely overly cautious, *red dots*/*lines* show participants who were on average extremely overly urgent, and *black dots*/*lines* show participants who were on average optimal. Different shades of *green/black* and *red/black* indicate intermediate values, with *dots* with more green/red indicating greater discrepancies from optimality from being too cautious/urgent. On the *y*-axis, positive values indicate overly cautious responding, negative values indicate overly urgent responding, and the *blue line* (0) indicates optimal responding. There appears to be a near-equal amount of subjects who are too urgent and too cautious, with many being near the line of perfect optimality

Taken together, the initial analyses and the model comparison analyses suggest an interesting pattern: Fig. 1 suggests that, after the first few blocks, participants are *on average* optimal, or very close to optimal, but the model comparisons suggest that free (non-optimal) threshold estimates are important. To investigate this more carefully, we performed the same analyses as above, but for each individual subject. The results for the first analysis—comparing estimated thresholds against the optimal threshold, across blocks of the experiment—are summarized in Fig. 2. This figure combines results across participants using Z-scores of the difference between the median of the posterior distribution for the threshold parameter from distribution of the optimal threshold parameter, with values above zero indicating too-cautious responding and values below zero indicating too-urgent responding. The results mirror the group-based analysis, with the average Z-score starting above zero (i.e., too cautious) and moving close to zero (i.e., optimal) over blocks. The most striking feature of the analysis, though, is the very large individual variation, with participants ranging from far too-urgent to far too-cautious, with these differences being centered on about 0 (i.e., optimality).

The second analysis compared the four model versions separately for each participant. Around 30% of the participants were best described by each of the too-cautious (*n* = 24), too-urgent (*n* = 24), and optimal (*n* = 21) models. The remaining 13% of participants (*n* = 10) were best fit by the freely-varying model.

## Discussion

Our study aimed to assess whether the previous findings of humans generally being overly cautious—when compared to the most efficient strategy—could be explained by methodological choices about the timing of the experiments. Previous studies assessing how human decision-strategies compared to the reward rate optimal strategy used fast task designs, where the participants’ responses were quickly followed by the next experimental trial. These fast designs cause the reward-rate-optimal strategy to be very urgent, and studies using these fast designs have mostly found participants to be more cautious than optimal (Simen et al., 2009; Balci et al., 2011; Evans & Brown, 2017; Starns & Ratcliff, 2012; Evans & Brown, 2017).

We introduced a long delay following incorrect responses, which makes the optimal strategy more cautious. As in other investigations, participants in our study began more cautious than optimal. During the second half of the experiment, the average speed–accuracy tradeoff setting was approximately optimal. More detailed analyses of individual participants showed that, on average, people were optimal, but there was substantial variation between people, with equally many participants adopting too-cautious as too-urgent strategies. These results suggest that the task design does have an impact on how close humans come to optimality. The impact was not as large as we hypothesized a priori, because our manipulations did not result in people adopting—on average—-too urgent speed–accuracy tradeoff settings. Our findings imply that previous studies may have concluded that humans behave more cautiously than optimal due to their settings of the experiments’ procedures (short delays between trials), and so generalizations about overly cautious strategies may not be warranted. Results reported by Simen et al., (2009), who manipulated RSI and interpreted their findings in terms of participants changing their strategy in qualitatively optimal manner, as well as Bogacz et al., (2010), who manipulated RSI and interpreted their findings in terms of most participants being overly cautious in general, are consistent with our explanation, as both of their results showed a decrease in the distance from optimality as RSI increased.

Our study also shows one of the first examples of a substantial proportion of participants (30%) adopting more *urgent* than optimal strategies (also see Malhotra, Leslie, Ludwig and Bogacz, 2017). Previous research in reward rate optimality has mostly made group-level inferences and has rarely shown evidence for overly urgent behavior. Our findings indicate that humans sometimes choose to be more urgent than optimal (without resorting to guessing). We also identified substantial variation between individuals in how close they come to optimality, and especially in which direction they are suboptimal (i.e., too urgent or too cautious). Interestingly, these findings are consistent with findings from a very different study, by Malhotra et al., (2017), who directly measured participants’ strategy using an expanded judgment task. They assessed whether strategies became more urgent as more time was spent on the decision (also known as “collapsing thresholds”; Ditterich 2006; Drugowitsch et al., 2012; Hawkins et al., 2015). When compared to an ideal observer model, Malhotra et al. found that in a slow-paced task participants were on average optimal in their urgency increase, with some increasing more than optimal (i.e., overly urgent behavior), but that in a fast-paced task participants were on average more cautious than optimal. Previous results that have only made group-level inference (e.g., Simen et al., 2009; Balci et al., 2011; Starns and Ratcliff, 2012; Evans and Brown, 2017) should be interpreted with caution, and future research should aim to better explore these individual differences.

Lastly, with our task we attempted to make the optimal strategy very cautious. This resulted in participants’ strategies being centered around optimal. On the other hand, earlier experiments have used tasks that make the optimal strategy very urgent, and these have resulted in participants’ strategies centered around overly cautious responding. The combination of these findings may suggest that although it is easy to push participants to being overly cautious with task design, it is much harder to make them overly urgent. An alternative explanation is that participants have a preferred speed–accuracy tradeoff policy (something close to the optimal strategy in our experiment) and it is difficult to push them far from this strategy. It will require future research investigating other manipulations (or tougher criteria) to disentangle these accounts. In addition, future research should also explore other experimental factors that may influence participant strategy, such as the task instructions provided.

## Footnotes

1 Our choice of the “simple” diffusion model over the “full” diffusion model (i.e., with between-trial variability parameters) was based upon findings indicating the simple diffusion to have superior recovery of parameters (Lerche & Voss, 2016).
